# Spatial Characteristics and Driving Factors of Provincial Wastewater Discharge in China

**DOI:** 10.3390/ijerph13121221

**Published:** 2016-12-09

**Authors:** Kunlun Chen, Xiaoqiong Liu, Lei Ding, Gengzhi Huang, Zhigang Li

**Affiliations:** 1 Faculty of Resources and Environmental Science, Hubei University, Wuhan 430062, China; XiaoqiongLiu_Hubu@163.com (X.L.); zhigangli@whu.edu.cn (Z.L.); 2 Regional Development and Environmental Response Key Laboratory of Hubei Province, Wuhan 430062, China; 3 Ningbo Polytechnic, Ningbo 315800, China; dinglei3616028@163.com; 4 Guangzhou Institute of Geography, Guangzhou 510070, China; 5 School of Urban Design, Wuhan University, Wuhan 430072, China

**Keywords:** wastewater discharge, LMDI, ESDA, spatio-temporal evolution, driving factor

## Abstract

Based on the increasing pressure on the water environment, this study aims to clarify the overall status of wastewater discharge in China, including the spatio-temporal distribution characteristics of wastewater discharge and its driving factors, so as to provide reference for developing “emission reduction” strategies in China and discuss regional sustainable development and resources environment policies. We utilized the Exploratory Spatial Data Analysis (ESDA) method to analyze the characteristics of the spatio-temporal distribution of the total wastewater discharge among 31 provinces in China from 2002 to 2013. Then, we discussed about the driving factors, affected the wastewater discharge through the Logarithmic Mean Divisia Index (LMDI) method and classified those driving factors. Results indicate that: (1) the total wastewater discharge steadily increased, based on the social economic development, with an average growth rate of 5.3% per year; the domestic wastewater discharge is the main source of total wastewater discharge, and the amount of domestic wastewater discharge is larger than the industrial wastewater discharge. There are many spatial differences of wastewater discharge among provinces via the ESDA method. For example, provinces with high wastewater discharge are mainly the developed coastal provinces such as Jiangsu Province and Guangdong Province. Provinces and their surrounding areas with low wastewater discharge are mainly the undeveloped ones in Northwest China; (2) The dominant factors affecting wastewater discharge are the economy and technological advance; The secondary one is the efficiency of resource utilization, which brings about the unstable effect; population plays a less important role in wastewater discharge. The dominant driving factors affecting wastewater discharge among 31 provinces are divided into three types, including two-factor dominant type, three-factor leading type and four-factor antagonistic type. In addition, the proposals aimed at reducing the wastewater discharge are provided on the basis of these three types.

## 1. Introduction

With the rapid growth of the economy and development of industrialization and urbanization [1,2], ecological deterioration has increasingly intensified. Thus, it has become a common focus around the world that every country promote the coordinate development between economic development and ecological environment [3]. According to the *China Environmental Quality Report*, among the 741 monitoring sections of China’s seven major river systems, the water quality of 41% monitoring sections barely meets the five standards, and 75% of all national lakes have developed eutrophication to different degrees [4,5,6], thus more than half of the residents have to drink contaminated water [7]. Due to the reality of increasingly severe water scarcity and water pollution, wastewater discharge and control has become the focus of the social and academic community. According to previous environmental statistical bulletins issued by the Ministry of Environmental Protection, the total wastewater discharge in China has witnessed a steady ascendance, and the domestic wastewater discharge has begun to exceed that of industrial wastewater and become the major source of the wastewater discharge since 1998. Therefore, under the dual pressures of industrial and domestic wastewater discharge, it is significant to scientifically and rationally recognize the spatio-temporal features and driving factors of wastewater discharge in the various regions to control the total emissions of pollutants and formulate regionally different emission reduction strategies [8] at present and in the near future.

Currently, the academic research is focused on two aspects of industrial wastewater discharge [9,10] and pollution control [11,12], namely its spatial-temporal features and influencing factors. Most research on the spatial-temporal characteristics has mainly paid attention to simple descriptions instead of relevant spatial analysis [13]. Sudan, for example, adopted the Equivalent Pollution Load Method to study the spatial-temporal variability of industrial wastewater emissions of Liaohe River in Liaoning Province [14]. Studies on the influential factors are relatively abundant, and primarily focus on the relationship between economic development and the wastewater discharge [15] from two aspects: one is to verify whether there exists an inverted U-shaped relationship between industrial wastewater discharge and economic growth as well as to ensure and predict the turning point of the industrial wastewater emission via Environmental Kuznets Curve (EKC) [16,17,18,19]; while another one is to study the correlated relationship between industrial wastewater discharge and economic growth via the Vector Auto-regression (VAR) model [20,21]. In recent years, studies on the decomposition of the driving factors of wastewater discharge have aroused much more attention. The Logarithmic Mean Divisa Index, for example, is widely adopted and continuously refined [22,23].

The existing studies have attained abundant achievements and developed profound understanding in various aspects, such as industrial wastewater discharge and its driving factors [24,25], the measurement of regional wastewater discharge [26], the characteristics of wastewater discharge in specific industries (for example, printing and dyeing) [27] or regions, the related relationships between wastewater discharge and economic development etc., thus to a certain extent, providing powerful support for a deep understanding of how to limit, restrict, assess and predict the water resources environment of urban, regional and even industrial development in the context of sustainable development. However, there still exist some research gaps in the cognition and research on total wastewater (including industrial and domestic wastewater) and the combination of its spatio-temporal distribution and driving factors. Confronted with the intensive pressures and challenges of the resource environment, the existing studies still need further improvement so as to comprehensively and incisively figure out this subject, among which studies such as comprehensive research on industrial and domestic wastewater discharge for a long time and on a nationwide basis, driving factors, differential analysis of measures and so on, help to comprehensively understand the limitations of the situation concerning the water resources environment during the decades of China’s rapid development.

Therefore, aiming at filling this gap, and utilizing the Exploratory Spatial Data Analysis (ESDA) method and the Logarithmic Mean Divisa Index (LMDI) method to analyze the spatio-temporal characteristics and driving factors underlying the changes in the provincial wastewater discharge in China, for this purpose, we selected the wastewater discharge in 31 provinces of China from 2002 to 2013 as research object, and account for its changed progress in general. Then, we reveal the spatio-temporal characteristics of wastewater discharge in different provinces and regions, and explore its driving forces by decomposing the factors that have affected the change of Chinese provincial wastewater discharge towards resource utilization efficiency, technological advancement, economy and population. Finally, some recommendations aimed at alleviating the wastewater discharge are proposed. We hope this study can provide a reference for controlling the total emissions of pollutants and formulating regionally different emission reduction strategies and discussing regional sustainable development and resources environment policies.

## 2. Data Sources and Research Methods

### 2.1. Data Sources

The total wastewater discharge data including industrial and domestic wastewater discharge per province in China, is acquired from the *China Statistic Yearbook on the Environment* [28] from 2002 to 2013. Considering the factors affecting wastewater discharge, this study uses the total water consumption, per capita GDP in China and total population as references, whose data come from the *China Statistical Yearbook* [29] and the official website of the National Bureau of Statistics of the People’s Republic of China [30]. After the adoption of the twelfth five-year plan, the wastewater being centrally processed became one type of the wastewater and took up small proportion of the total wastewater. Therefore, this study does not include wastewater being centrally processed as the research object. Meanwhile, due to the lack of statistical data, this study does not analyze the following regions: Hong Kong Special Administrative Region, Macao Special Administrative Region, Taiwan, Diaoyu Island, Sansha City and the South China Sea Islands.

### 2.2. Research Methods

#### 2.2.1. Exploratory Spatial Data Analysis (ESDA) Method

Characteristics of the spatial distribution of wastewater discharge in every province are measured via the Exploratory Spatial Data Analysis (ESDA) method which describes the spatial distribution of the provincial wastewater discharge. Which utilizes the global spatial autocorrelation analysis and local spatial autocorrelation analysis.

(a) Global Spatial Autocorrelation. It reflects the agglomeration of the research object in the whole space. The index of the Moran’s *I* is calculated to reflect the spatial agglomeration and its correlation. The formula is:
  (1) $$I=\frac{n\sum_{i}\sum_{j}W_{ij}\left(x_{i}-\bar{x}\right)\left(x_{j}-\bar{x}\right)}{\left(\sum_{i}\sum_{j}W_{ij}\right)\sum_{i}{\left(x_{i}-\bar{x}\right)}^{2}}$$
  in the formula, n represents the total number of research areas; $x_{i}$ and $x_{j}$ represents the total wastewater in area i and area j respectively; $\bar{x}$ is the annual average value of wastewater in different provinces. $W_{ij}$ represents the spatial weight matrix, which is calculated by the nearest neighbor classification algorithm. I ranges from −1 to 1. If I is less than 0, it indicates that the relationship among wastewater of different provinces is spatially negative and shows a strong spatial heterogeneity; if I exceeds 0, it indicates that the relationship among the wastewater of the different provinces is spatially positive and the wastewater discharge of different provinces is spatially intensive, which means it exerts certain effects among one another; if I equals 0, it shows that the distribution of the provincial wastewater is random. The significance is measured by the Monte Carlo method of the Geoda software. Indicators of feedback include *E*(*I*) (the value of the mathematical expectation), representing an expected value of a scatter random variable, which is the summation of the probability of each possible outcome in the test; *Sd.* (the standard deviation) representing the arithmetic square root of variance, which reflects the discrete degree between individuals in the group; *P*(*I*) (the significance level)representing the probability of error that population parameter falls beyond a certain area.
(b) Local Spatial Autocorrelation. It reflects the spatial difference of the wastewater in the whole country, but it is difficult to show the spatial differences among provinces. The interaction among provinces close to each other should be measured by these methods, which include the Moran scatter diagram and the statistic of the Local Moran’s I. The Moran scatter diagram describes the correlation between the variable and the lagging vector, which shows the degree of correlation and differentiation among the value of the spatial unit. The diagram consists of four quadrants, including high-high (HH) type, high-low (HL) type, low-high (LH) type and low-low (LL) type. These four types represent four kinds of relationship between wastewater discharge of one province and that of its neighboring provinces respectively. HH type indicates that wastewater discharge of one province and wastewater discharge of its surrounding provinces are very high (the relationship between them is spatially positive). HL type indicates that wastewater discharge of one province is high but wastewater discharge of its surrounding provinces is low (the relationship between them is spatially negative and the difference is significant). LH type indicates that wastewater discharge of one province is low but that of its neighboring provinces is high (the relationship between them is spatially negative and the difference is significant) and LL type indicates that wastewater discharge of one province and that of its neighboring provinces are very low (the relationship between them is spatially positive).

#### 2.2.2. Logarithmic Mean Divisia Index (LMDI) Model

The index decomposition analysis method, first put forward by several economists in the 1980s [31], decomposes a study object into several sub-items, and analyzes the influence of each item on the study object.

After years of improvement and research, the DI index decomposition analysis method and Laspeyres index decomposition analysis method were put forward. LMDI represents Logarithmic Mean Divisia Index method in DI’s index decomposition analysis method, and it is a digital model produced by Ang and his team [32,33,34]. This model was used to learn more about the contribution of people’s activities to energy consumption and pollution discharge [35,36]. It was proposed to solve the problem of residual term that the previous weighted average cannot deal with. After a period of study, Ang and his team solved the problem of zero and negative values, which enables the Logarithmic Mean Divisia Index model to be suitable for all problems in factor decomposition. Therefore, it is widely used in analyzing driving factors.

This study decomposes the influential factors of the wastewater discharge by the LMDI method and analyzes distribution of resource utilization efficiency, technological advance, economy and population to the discharge of the wastewater. At first, the Kaya identical equation [37,38,39] is used to decompose wastewater:

(2) $$W^{t}=\sum_{i}^{n}D_{i}^{t}=\sum_{i}^{n}\left[\left(\frac{W_{i}^{t}}{C_{i}^{t}}\right)\cdot \left(\frac{C_{i}^{t}}{G_{i}^{t}}\right)\cdot \left(\frac{G_{i}^{t}}{P_{i}^{t}}\right)\cdot P_{i}^{t}\right]=\sum_{i}^{n}\left(W_{i,eff}\cdot W_{i,tec}\cdot W_{i,eco}\cdot W_{i,pop}\right)$$

$W^{t}$ represents the total wastewater discharge in years t and $C_{i}$ represents the total domestic wastewater discharge in area i. $G_{i}$ represents the gross regional product and $P_{i}^{t}$ represents the total population in area i.

$W_{i,eff}$ represents the efficiency of resource utilization and is indicated by the wastewater produced by the total consumption of every unit of water; every unit of water consumption represents the efficiency of the energy utilization. The ratio is lower, the more efficient the energy is. Meanwhile, the change of this ratio will affect wastewater discharge.

$W_{i,tec}$ represents technological advance and is shown by the total consumption of every unit of water resource. The lower the ratio is, more effective the technology is.

$W_{i,eco}$ represents the economy, that is per capita gross social product, and is indicated by GDP. The economic development should affect wastewater discharge.

$W_{i,pop}$ represents the population, and indicates the influence of the population on wastewater discharge.

Based on the LMDI method, the formulas indicating the contribution of every factor to wastewater discharge are as follows:

(3) $$\Delta W_{i,eff}=\frac{W_{i}^{t}-W_{i}^{0}}{\ln W_{i}^{t}-\ln W_{i}^{0}}\cdot \ln\left(\frac{W_{i,eff}^{t}}{W_{i,eff}^{0}}\right)$$

(4) $$\Delta W_{i,tec}=\frac{W_{i}^{t}-W_{i}^{0}}{\ln W_{i}^{t}-\ln W_{i}^{0}}\cdot \ln\left(\frac{W_{i,tec}^{t}}{W_{i,tec}^{0}}\right)$$

(5) $$\Delta W_{i,eco}=\frac{W_{i}^{t}-W_{i}^{0}}{\ln W_{i}^{t}-\ln W_{i}^{0}}\cdot \ln\left(\frac{W_{i,eco}^{t}}{W_{i,eco}^{0}}\right)$$

(6) $$\Delta W_{i,pop}=\frac{W_{i}^{t}-W_{i}^{0}}{\ln W_{i}^{t}-\ln W_{i}^{0}}\cdot \ln\left(\frac{W_{i,pop}^{t}}{W_{i,pop}^{0}}\right)$$

The above four formulas represent the influence of efficiency of the water resource utilization, technological advance, economy and population on the amount of wastewater discharge. If the result is positive, it means the growth of these factors will increase the wastewater discharge. If the result is negative, it means the reduction of these factors will control wastewater discharge.

## 3. The Spatio-Temporal Features of Wastewater Discharge

### 3.1. The Temporal Evolution of the Total Wastewater in China

**Figure 1:**
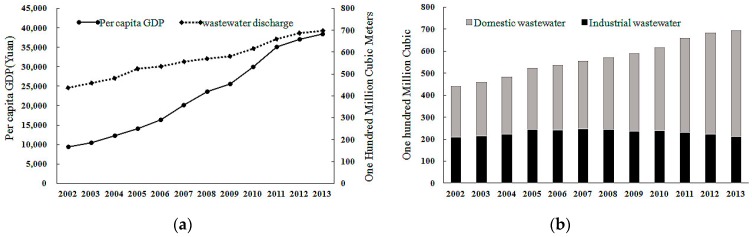
Wastewater discharge and the change of per capita GDP in China, 2002–2013. ((**a**) represents the per capita GDP and total wastewater discharge of China from 2002 to 2013, while (**b**) represents domestic and industrial wastewater discharge of China from 2002 to 2013).

Figure 1, showing the per capita GDP and total wastewater discharge in China, indicates that with the economic development, the total wastewater discharge has steadily increased. During the 12 years examined, wastewater discharge increased by 5.6 billion cubic meters. According to the growth rate of the wastewater discharge per province, three phases can be described on the basis of the change of the total wastewater discharge from 2001 to 2013: the first one is from 2002 to 2005, when the growth rate of the national wastewater was below 6%; the second one is from 2005 to 2009, when the growth rate remained at 3.6% and increased slowly. The third one is from 2009 to 2013, when the growth rate was 6.4% and it increased more and more quickly. As shown in Figure 1, wastewater discharge is closely related with economic development, and its upward trend is in accordance with that of the economic development.

**Figure 2:**
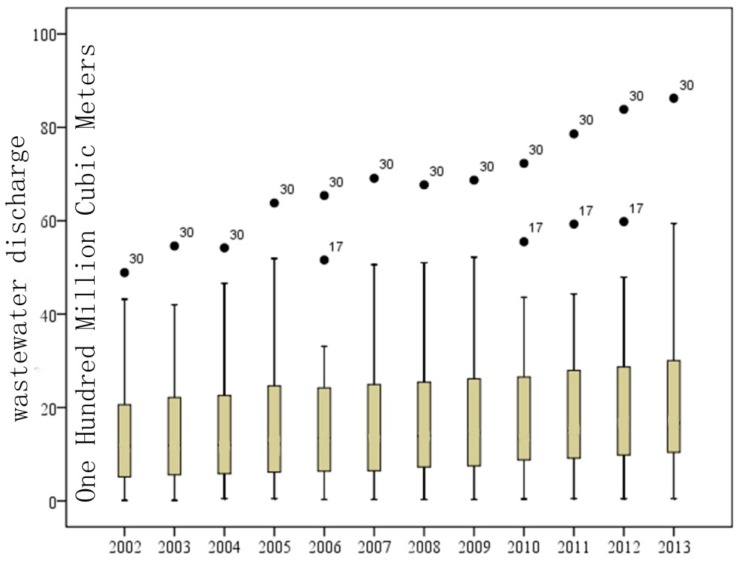
Provincial inter-annual variability of the wastewater discharge in China, 2002–2013 (the upper limit and the lower limit of each box are within the range of the normal value. The two ends of the rectangle box correspond with four quantizes, the line in the middle is the median; Thirty and seventeen are the abnormal points, representing Guangdong Province and Jiangsu Province respectively).

To further analyze differences in the wastewater discharge during distinct years, the boxplot graph (Figure 2) is drawn to describe the inter-annual wastewater discharge differences among the 31 provinces. Generally, the maximum value of the wastewater discharge in every province and city increased and the minimum value changed a little during the research period. It means that the extreme value of wastewater discharge increased, which indicates that the absolute difference of wastewater discharge among provinces kept increasing; the total wastewater discharge during the research period of Guangdong Province was abnormal, and it became the province whose amount of wastewater discharge is the biggest in the whole country; Jiangsu Province ranked the second, becoming abnormal in 2006 and from 2010 to 2012. Wastewater discharge in almost every province increased, except in some regions such as Beijing City and the Tibet Autonomous Region, whose amount of wastewater discharge decreased in certain years.

### 3.2. The Spatial Distribution Change of the Provincial Wastewater Discharge

**Figure 3:**
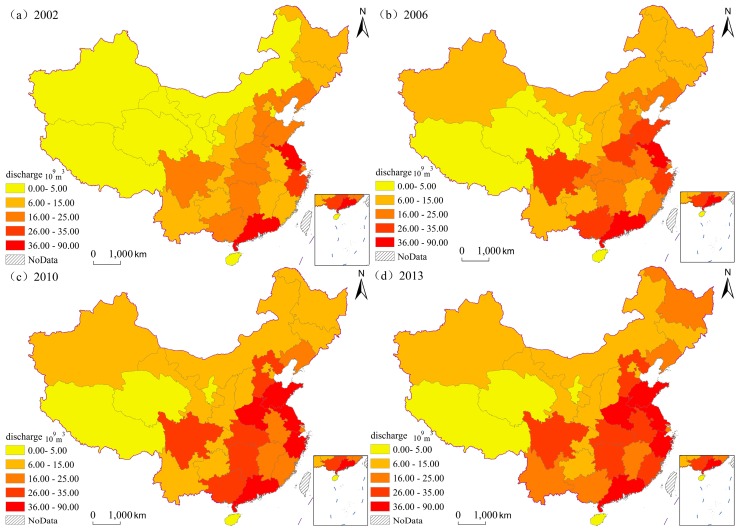
Distribution of wastewater discharge at national level in China, 2002–2013.

To further discuss the spatial relationships of wastewater discharge among provinces and clarify the format of the spatial evolution of wastewater discharge, this study divides the wastewater of 31 provinces from 2002 to 2013 into five types, including the low discharge ranging from 0 to 0.5 billion cubic meters, the low and middle discharge ranging from 0.5 to 1.5 billion cubic meters, the middle discharge ranging from 1.5 to 2.5 billion cubic meters, the middle and high discharge ranging from 2.5 to 3.5 and the high discharge ranging from 3.5 to 9 billion cubic meters, on the basis of the natural breakpoint method. Four years, that is 2002 (the first year of the study), 2006 (the first year of the eleventh five-year plan), 2010 (the last year of the eleventh five-year plan) and 2013 (the last year of the study), are selected to analyze and depict the figure of the general spatial distribution (Figure 3).

Generally, from 2002 to 2003, the wastewater of almost all provinces increased and the level of wastewater discharge rose as well. The number of provinces with high level increased, from Jiangsu Province and Guangdong Province in 2002 to Jiangsu Province, Guangdong Province, Shandong Province, Zhejiang Province and Henan Province in 2013. From the distribution characteristics of wastewater, the provinces with high wastewater discharge mainly include Jiangsu Province in the east, Guangdong Province in the south and Sichuan Province in the west. Provinces with low discharge of wastewater mainly include the Xinjiang Uygur Autonomous Region, Qinghai Province and the Tibet Autonomous Region in the west. The boundary between provinces with high discharge and provinces with low discharge is obvious, almost parallel with the population boundary named the Hu Huanyong line. These two areas show the obvious spatial relativity. From the dynamic perspective, the area with high discharge expanded. The grade of wastewater discharge in Anhui, Fujian, Shandong, Henan and Inner Mongolia increased. The newly added areas with high discharge continuously spang up, expanding from the east to the mid-west.

### 3.3. The Spatial Variation of the Provincial Wastewater Discharge

The spatial relationship among provinces is described through calculating the total wastewater from 2002 to 2013 and the index of Moran’s *I*. The results are shown below.

Table 1 shows that the index of Moran’s *I* increased from 0.2182 in 2002 to 0.2842 in 2011 at its peak, then it decreased to 0.2519 in 2013, which passed the 5% significance test. The value of the index of the Moran’s *I* remains positive, ranging from 0.2182 to 0.2842. These values show a stable positive spatial correlation, and indicate that total wastewater in China is markedly spatially concentrated during the research period.

**Figure 4:**
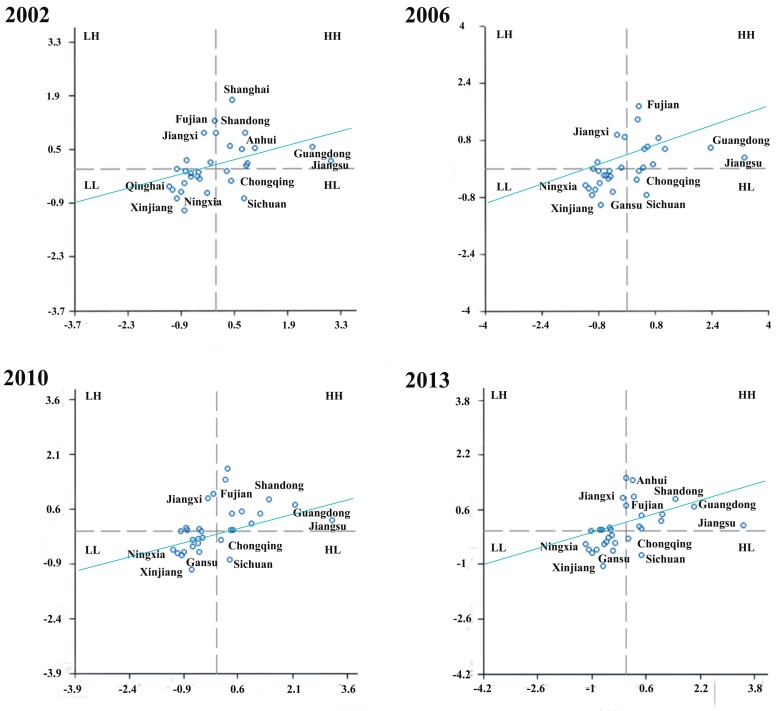
Moran scatter diagram of the provincial wastewater discharge in China, 2002–2013.

To further analyze the spatial agglomeration of the wastewater discharge and search for areas which discharge wastewater intensively and need to be controlled, this study uses the GeoDa software (Environmental Systems Research Institute, Redlands, CA, USA) to establish the Spatial Weight Matrix and find out the spatial agglomeration in the partial areas. Finally, the figure of the spatial agglomerative distribution and the Moran scatter diagram in 2002, 2006, 2010 and 2013 can be drawn (Figure 4).

The Figure 4 indicates that: (1) areas belonging to the HH type gathered in the eastern coastal region. During the research period, the HH areas include Shandong Province and Anhui Province in 2002 and then Fujian Province is added in 2013. The eastern coastal region is economically developed and discharges more wastewater than other regions; (2) areas belong to the HL type are distributed in Sichuan Province and Chongqing City during the research period, and around them are provinces discharging less wastewater such as Gansu Province, the Tibet Autonomous Region and Yunnan Province; (3) Jiangxi Province belongs to the LH type because it is near the provinces discharging more wastewater, such as Guangdong Province and Fujian Province. Fujian Province and Anhui province generally belong to the HH type, but they also conform to the LH type in some years; (4) provinces in northwest China belong to the LL agglomeration type, such as the Xinjiang Uygur Autonomous Region, Gansu Province and the Ningxia Autonomous Region. In 2003, Qinghai Province and Inner Mongolia are added. At present, areas belong to the LL type are mainly distributed in the arid and semi-arid regions, coincident with areas of low wastewater discharge.

## 4. Analysis of the Driving Factors of Wastewater Discharge

The above analysis indicates that the total amount of the provincial wastewater decreased from southeast China to northwest China. On the basis of the LMDI model, this study has classified the driving factors of wastewater discharge into four types, including the efficiency of the resource utilization, the effect of the technological advance, the effect of the economy and population .Thus, this study further discusses the change of the driving factors of wastewater discharge.

**Figure 5:**
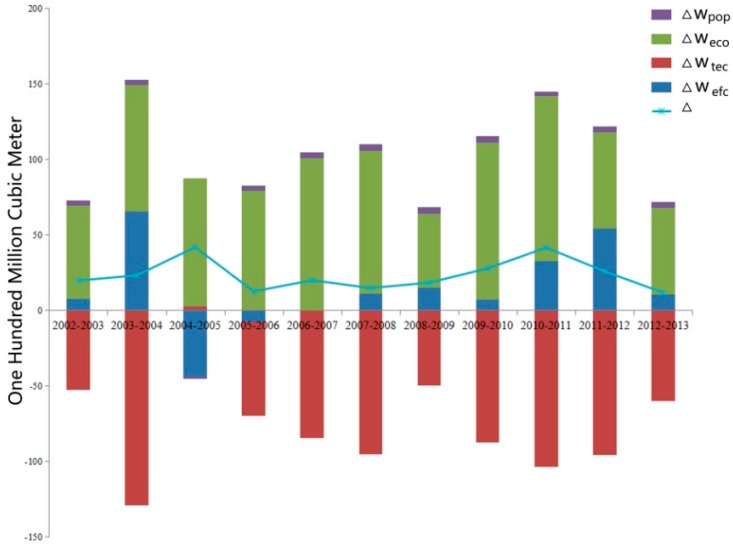
Decomposition analysis results of wastewater discharge in China, 2002–2013.

Within the whole nation, the economy is the dominant driving factor and displays a positive relationship with wastewater discharge. From 2002 to 2007, the contribution value of the economy continuously increased. However, it slumped from 2007 to 2009 and grew dramatically after 2009. In 2011, it reached a peak, followed by a decline. The contribution value of economic development is at least 4.9 billion cubic meters and at most 10.4 billion cubic meters. The average contribution value reached 8 billion cubic meters per year. Technological advance reduced the wastewater continuously except the years from 2004 and 2005, and plays an important part in wastewater discharge. It indicates that the technological advances prevent the wastewater from increasing. Indeed, technological advances decreased the high discharge of the wastewater due to the unsound economic development; the contribution value of the efficiency of the resource utilization is not stable. In most years, the value was positive while it was negative in a few years. The efficiency of the resource utilization obviously affected the wastewater discharge in certain years. The population exerts a less important effect on the wastewater discharge and its effect is relatively stable. From Figure 5, it can be seen that the wastewater discharge had ups and downs but displayed an upward trend. From 2002 to 2005, the value continuously increased. However, from 2005 to 2006, it slumped and then steadily grew from 2011 to 2013. The reason for the decline is that a plan of energy reduction and emission reduction was adopted in the twelfth five-year plan, which aimed to establish a water conserving society.

**Figure 6:**
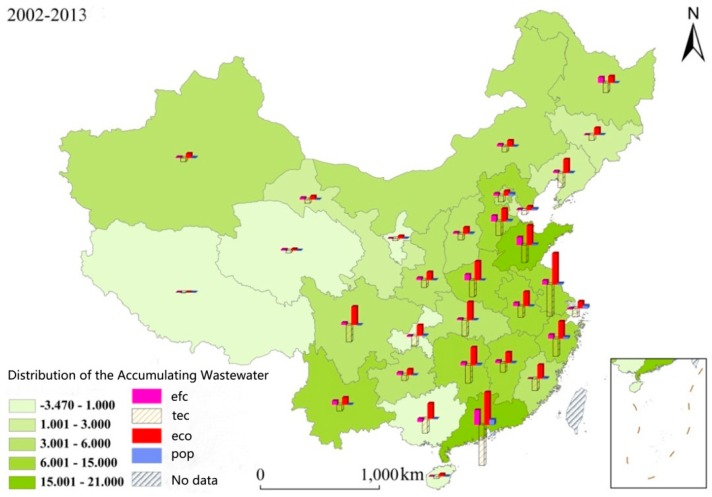
The distribution chat of the accumulated variation and index contribution of provincial wastewater discharge in China, 2012–2013.

To study the driving factors of provinces in detail, the index contribution value of each province was obtained through LMDI, the index contribution of each province is calculated, and the distribution chart of the contribution in all provinces is drawn via ArcGIS10.0. At the same time, the index of each province is overlapped in Figure 6, which could conspicuously show the index contribution value of each province.

Generally, the wastewater spatially decreased from the southeast to the northwest. The provinces with the high increase of the wastewater discharge mainly gather in the economically developed areas, such as Guangdong Province and Jiangsu Province. Provinces with less increase of wastewater discharge mainly gather in the west arid regions, such as the Tibet Autonomous Region and Qinghai Province.

### 4.1. The Effect of the Driving Factors on the Added Value of the Wastewater Discharge

#### 4.1.1. The Effect of the Economy of Scale on the Value Added of Wastewater Discharge

The regional economic development is the dominant driving factor, affecting the wastewater discharge [25]. From Figure 6, it can be seen wastewater increased around the nation due to the economic development. The added value of wastewater in these provinces, such as Jiangsu Province, Shandong Province, Henan Province, Hubei Province, Hunan Province and Sichuan Province, exceeds three billion cubic meters. The added value of wastewater in Guangdong Province and Jiangsu Province, which have abundant water resources and are economically developed, exceeded five billion cubic meters. However, economic development could not be at the expense of the environment. Because the economic development of the above provinces increased the discharge of wastewater which has heavily destroyed nature and the residential environment. The balance between the economic development and the environmental protection should be emphasized. Meanwhile, the economic development of these provinces, such as Hainan Province, Qinghai Province, Tibet Autonomous Region and Ningxia Autonomous Region, is slow. Therefore, the wastewater of these provinces which does not exceed 0.5 billion cubic meters did not increase significantly. However, the regional economic development differs widely in distinct places. In the midwest of China, the economy continues to develop, therefore, according to the specific situation of each province, the wastewater will increase, so standards for wastewater discharge should be set up. Measures for reducing wastewater should be earnestly implemented and the associative supervision for wastewater discharge and the punishment should be further enhanced.

#### 4.1.2. The Effect of Technological Advances on the Added Value of Wastewater Discharge

Generally speaking, technological advances could control wastewater discharge and exert significant effects on it. Through the whole country provinces, including Jiangsu Province, Zhejiang Province, Henan Province, Hubei Province, Hunan Province, Guangdong Province and Sichuan Province, have significantly controlled the wastewater by improving the production technology and the wastewater discharge technology. The technological advances have reduced wastewater discharge up to at most 7 billion cubic meters and at least 2.8 billion cubic meters. Guangdong Province and Jiangsu Province with high technology and rapid economic development, have reduced the wastewater by 5.5 billion cubic meters and 7 billion cubic meters, respectively. However, the effect of technological advances is not significant in areas such as Hainan Province, the Tibet Autonomous Region and the Ningxia Autonomous Region. On the one hand, the wastewater discharge of these three listed areas is not high. On the other hand, the economic development and the technological development is limited. Generally, the effect of technological advances on the reduction of wastewater is gradually weakened from the east to the west. This shows that science and talents should be emphasized, under the policy of the Rise of the Central China and the West Development [40,41]. The local environmental protection via technological advances should be promoted, so that the wastewater reduction technology could be improved in central and western China.

#### 4.1.3. The Effect of the Efficiency of the Water Resource Utilization on the Added Value of Wastewater Discharge

The efficiency of the water resource utilization plays an increasingly important role in wastewater discharge [42,43,44]. From the viewpoint of the whole country, the wastewater of most provinces increased, because of the low efficiency of the water resource utilization. Provinces and cities including Shanghai City, Guangxi Province and Chongqing City, reduced their wastewater by improving the efficiency of their water resource utilization. The wastewater of Guangdong Province and Shandong Province increased significantly, reaching 2.6 billion cubic meters and 1.4 billion cubic meters, respectively, because of the low efficiency of the water resource utilization. These two provinces are economically developed and possess plenty of water. At the same time, provinces and cities, including Jilin Province, Shanghai City, Fujian City, Guangxi Province, Hainan Province and Chongqing City, controlled their wastewater by improving the efficiency of their water resource utilization. The wastewater of these provinces and cities didn’t decrease much. This shows that people should vigorously advocate water conservation and improve the efficiency of the water resource utilization and the industrial reuse of the water resources as well [45].

#### 4.1.4. The Effect of the Population on the Added Value of Wastewater Discharge

The effect of the population on the wastewater discharge was not significant in each decomposition index. The wastewater of Shanghai City and Guangdong Province, which have the most immigrants, was relatively high and increased to 0.46 billion m^3^ and 0.9 billion m^3^, respectively. In other provinces, the wastewater was below 3.5 billion cubic meters. In Anhui Province, Sichuan Province and Guizhou Province, the population even played a negative role in increasing wastewater. For example, the population of Guizhou Province decreased from 38.37 million in 2002 to 35.02 million in 2013. During the past twelve years, it has decreased by 3.35 million. Because of the reduced population, it is important to pay more attention to the reduction of the wastewater discharge. As one of the fundamental state policies, population targets were adjusted recently [46,47], but it could remain unchanged for a long time. Therefore, suggestions on the population will not be given. With the gradual increase of the domestic wastewater year by year, it accounts for the largest proportion of the total wastewater. Therefore, it is necessary to improve and enhance people’s awareness of water conservation so as to reduce wastewater at the source.

### 4.2. Recommendations for Reducing Wastewater Discharge Based the Spatial Difference of the Driving Factors

According to the model, the absolute accumulated value of the decomposition factors from 2002 to 2013 and the total absolute contribution value are calculated. The main driving factors affecting wastewater discharge are identified. Then the method of minimum variance is used to compare the actual distribution of the contribution with the theoretical distribution, and the minimum variance is calculated. Finally, the results will be imported into the statistical software SPSS (International Business Machines Corporation, Armonk, NY, USA), and cluster analysis conducted. Then the main driving factors should be sorted out on the basis of the structural similarity and difference, so as to spatially classify the driving factors.

**Figure 7:**
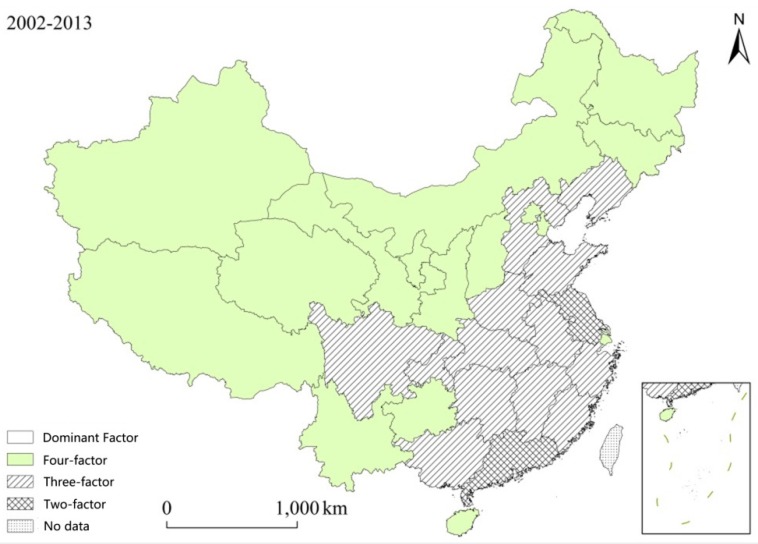
Types of the main driving factors of the wastewater discharge in China.

From Table 2, it can be seen that the driving factors which affect the spatial changes of wastewater discharge can be divided into four types. The dominant driving factors of two provinces are the economy and technological advances. Both of the driving factors play a significant effect on wastewater discharge (two-factor dominant type).The main driving forces of thirteen provinces are efficiency of the water resource utilization, the economy and technological advances (three-factor leading type); The dominant driving factors of sixteen provinces are efficiency of the water resource utilization, the economy, technological advances and population (four-factor antagonistic type). The driving factor result are shown in Figure 7.

#### 4.2.1. Two-Factor Dominant Type

The two-factor dominant type depends on the economy and technological advances. Two provinces, Jiangsu Province and Guangdong Province, belong to this type. These two provinces are economically developed. These two factors including the economy and technological advances are the dominant factors which act on the increase of wastewater discharge.

For example, Guangdong Province is economically developed. Its per capita GDP increased from 15,361 yuan per person in 2003 to 58,833 yuan per person in 2013. Wastewater increased by 8.2 billion cubic meters due to the economic development and improper utilization of the water resources from 2002 to 2013; during the twelve years, the new projects added every year could process the wastewater from 135,000 cubic meters per day to 200,000 cubic meters per day. The improved technology and measures of the wastewater reduction controlled the increase at 7 billion cubic meters of the wastewater. It shows that cities belong to the two-factor dominant type are developed but their economy is extensive. Because the economic development has brought about a series of the serious problems, it should cost a lot to deal with these problems. These economically developed provinces, on the one hand, should bear more responsibility for reducing wastewater. They need to control the total wastewater through the policy of paying for wastewater discharge and deal with this problem by taking market-oriented measures [48,49]; On the other hand, based on the policy of transferring the secondary industry and the labor force at the beginning of the thirteenth five-year plan, these provinces should not only keep their economy increasing but also achieve the goal of reducing wastewater. When the secondary industry is transferred, technologies and talents should be provided for the surrounding areas so as to support their projects on the reduction of the wastewater discharge and maintain the regional coordinated development.

#### 4.2.2. Three-Factor Leading Type

The three-factor leading type also depend on the economy and technological advances. Their effect was weakened while the efficiency of the water resource utilization was enhanced. Provinces and cities belonging to this type are mainly located in the south of the Yangtze River, including Hubei Province, Sichuan Province, Zhejiang Province, Hunan Province, Shandong Province, Henan Province, Jiangxi Province, Chongqing City, Liaoning Province, Fujian Province, Anhui Province, Hebei Province and Jiangxi Province.

In these areas, the efficiency of the water resource utilization had both positive and negative effects on wastewater discharge. For example, the economy of Fujian developed well, with its per capita GDP reaching 13,497 yuan per person in 2002 to 57,856 yuan per person in 2013. During the research period, wastewater increased to 2.37 billion cubic meters due to the economic development. During the twelve years, the new projects added every year could process wastewater from 111,000 cubic meters per day to 284,000 cubic meters per day. The efficiency of the water resource utilization increased from 62.6 yuan per cubic meter to 205.5 yuan per cubic meter. The reduced wastewater due to the improved efficiency of the water resource utilization and technological advances greatly offset the increase of the wastewater due to the economic development and population growth. Therefore, wastewater of Fujian Province was controlled. The added value of the wastewater of Anhui Province due to the economic development and technological advances is the same as that of Fujian Province, but the efficiency of the wastewater utilization is still lower. The efficiency of the water resource utilization of Anhui Province was 13.7 yuan per cubic meter less than that of the Fujian Province in 2002 and 58.9 yuan per cubic meter less than that of the Fujian Province in 2013. Wastewater was increased by 0.53 billion cubic meters due to the low efficiency of the water resource utilization. Generally, the added value of the wastewater is far more than that of Fujian Province. For this type of provinces, the recycling rate of the wastewater should be increased. The efficiency of the water resource utilization could be improved by restricting the water quotas, charging at different levels, building the index of the water consumption that one ten thousand yuan results in and including the achievement of water consumption tasks into the achievement evaluation of the local government [50].

#### 4.2.3. Four-Factor Antagonistic Type

The four-factor antagonistic type depends on the efficiency of the water resource utilization, the economy, technological advances and the population. Provinces and cities belonging to this type are mainly located in western region of China, such as Hainan Province, the Ningxia Autonomous Region, Qinghai Province, the Tibet Autonomous Region, Tianjin City, the Xinjiang Xinjiang Uygur Autonomous Region, Gansu Province, Yunnan Province, Shanxi Province, Inner Mongolia, Guizhou Province, Shanxi Province, Beijing City, Jilin Province, Shanghai City and Heilongjiang Province. Under the four types of driving factors, the growth of the provincial wastewater offsets the decease of the provincial wastewater.

There are two kinds of cities in this type of driving factors. One is the developed cities such as Beijing and Shanghai. For example, the economy of these two cities has rapidly developed. The per capita GDP increased from 35,329 yuan per person in 2002 to 90,993 yuan per person in 2013, with an average annual growth rate reaching 10%. With the economic developing, wastewater discharge increased. Meanwhile, the domestic wastewater discharge increased from 1.27 billion cubic meters in 2002 to 1.772 billion cubic meters in 2013; at the same time, the efficiency of wastewater utilization and the technology of the water conservation increased. The efficiency of wastewater utilization increased from 62.2 yuan per cubic meters to 203.6 yuan per cubic meters. During the research period, the new projects added could process the wastewater from 111,000 cubic meters per day to 280,000 cubic meters per day, reducing wastewater by over 1.65 billion cubic meters. This could offset the growth of the wastewater which was produced by the economic development and population growth. Generally, the discharge of wastewater was reduced in Shanghai. Another situation is the case of underdeveloped provinces such as Guizhou and Gansu. Although Guizhou Province is underdeveloped, its per capita GDP increased from 3000 yuan per person in 2002 to 23,151 yuan per person in 2013, with a growth rate reaching 16%. The economic development improved the efficiency of the water resource utilization and the technology. During the research period, the efficiency of the water resource utilization increased from 34.62 yuan per cubic meters to 186.2 yuan per cubic meters. The new projects added every year could process the wastewater discharge from 111,000 cubic meters per day to 560,000 cubic meters per day. The improved efficiency of water resource and technological advances controlled the wastewater. For the developed provinces, the science and technology should be enhanced, the technology of processing the wastewater should be innovated and foreign advanced technology and management experience should be introduced. Through these methods, the technology of processing the wastewater could be improved; for the underdeveloped provinces, wastewater discharge could be reduced by the measures resulting from policies such as the mechanism for ecological compensation.

## 5. Conclusions

With the population growth, economic development and the promotion of urbanization and industrialization, it becomes an increasingly important part to improve the water environment and reduce water pollution for the ecological development of civilization in China [51]. From the perspective of spatial analysis and factor decomposition, this study aimed at, by taking the total wastewater discharge from 31 provinces and cities in China from 2002 to 2013 as the research object, discussing the corresponding policies concerning wastewater reduction through the understanding of its spatio-temporal features and driving factors so as to provide references for establishing national macroeconomic policies. Through this study, the following conclusions can be obtained:

(a) The spatio-temporal characteristics of wastewater discharge in China

The temporal features of wastewater discharge show a steady upward trend during the research period, and the domestic wastewater, as the dominant source of wastewater, is higher than industrial wastewater. At the same time, wastewater discharge demonstrates a significant positive correlation with space. Areas with high discharge are concentrated in the economically developed coastal provinces such as Jiangsu Province, Guangdong Province, Shandong Province and Zhejiang Province. These provinces are the primary control areas of wastewater reduction. Areas with high wastewater discharge tend to be transferred to the northwest, which indicates that, with further promotion of the Rise of Central China and the Western Development Program, the rapid development of the economy in the central and western China should be accompanied by a growth in wastewater discharge. Water resources and the water environment in the central and western regions will confront intensive pressure.

(b) The driving forces of changes of wastewater discharge in China

Based on the LMDI method, the driving factors of Chinese domestic wastewater discharge are divided into four types: resource utilization, technological advances, the economy and the population. The economy and technological advances are the dominant factors for the wastewater discharge, and play a significant role in it. The economic development leads to an increase in wastewater and exerted a decisive effect in increasing it, while technological advances control the increase of wastewater discharge and is one of a crucial factors among all the inhibitory factors; water utilization efficiency plays a destabilizing role on wastewater discharge, which means in most provinces, low efficiency of water resource utilization results in the increase of wastewater discharge whereas in a few provinces the improved efficiency of water resource utilization reduces the wastewater discharge; population effect, among the four decomposition factors, is the least important one for wastewater discharge. However, the population can also affect the local wastewater discharge to a certain extent in some provinces where the amount of population is too large or too small.

(c) Suggestions for controlling wastewater discharge in China

Via the minimum variance method, the different types of all driving factors from 31 provinces in China are divided into two-factor dominant type, three-factor leading type and four-factor antagonistic type, which provides a comparable figure to factors affecting wastewater discharge in every region and more directly reflects the spatial distribution features of the factors affecting China’s wastewater discharge. Meanwhile, comprehensive research at home and abroad, with China’s actual situation, the suggestions for controlling water pollution are as follows: (1) comprehensively controlling wastewater discharge, establishing an inter-provincial linkage mechanism in the economically developed coastal provinces where high wastewater discharge is concentrated, making neighboring provinces share the common responsibility of reducing the wastewater and, jointly preventing and controlling the wastewater; in the process of industrial introduction, the central and west provinces should set up strict environmental standards, and restrict the development and introduction of industries with high water consumption and high wastewater discharge. While introducing industries, the supporting facilities and related technologies should be focused on [52,53]; (2) when measuring socio-economic development, provinces should introduce and inform the concept of a green GDP index, associate the performance of water conservation goals with local government performance assessment, and ensure wastewater reduction while the national economy maintains a sound development; further strengthening the scientific and technological support, innovating wastewater processing technology, training of wastewater treatment personnel [54], introducing foreign advanced wastewater treatment and management methods, and improving wastewater treatment technologies; promoting the industrial intensive development, improving the efficiency of resource utilization and promoting wastewater recycling by restricting the water quotas, charging at different levels [53], and establishing water consumption indicators based on ten thousand yuan GDP and other ways; vigorously advocating water reservation, communication and popularization of the knowledge of how to deal with domestic wastewater, enhancing public awareness of water conservation, strengthening the public participation and social supervision, and reducing the wastewater discharge at the source.

(d) Experiences and further research directions

Confronted with the intensive pressure and challenges of the resources environment, and based on the existing research, combining with the ESDA method and the LMDI method, this paper studies the spatio-temporal distribution characteristics and driving factors of wastewater discharge in China, and makes up for this problem to some extent. From the point of view of the research methods, the ESDA method focuses on the use of spatial expression to reflect the spatial pattern of the existence and evolution of things, while the LMDI method focuses on explaining the dominant factors that contribute to changes in things through the screening of the impact factor intensity measures. The combination of the two methods, in the visual expression of evolutionary pattern of things and the dominant factors on the basis of cohesion, is conducive to the different stages of the driving factors of the spatial relationship between the different units and the formation of the dominant factor pattern more intuitive and in-depth understanding, which is of great significance for the study of energy saving and emission reduction on the larger spatial scale, such as watersheds, regions or countries, where the land area is vast and the regional differences (including the differences in natural conditions and socio-economic differences) is obvious. Wastewater reduction measures are also highly targeted according to this method. Meanwhile, in China, as the largest developing country as well as the fastest-growing developing country, the wastewater discharge and environmental issues are of great significance for other developing countries, so this study not only helps to understand China’s problem of wastewater emissions, but also provides a certain reference for other developing countries facing similar problems. In addition, based on the shortcomings in the field of wastewater research, the authors hope to improve the research methods for studying China’s wastewater discharge problems, using a smaller scale (for example, prefecture-level cities and county-level cities).

## Figures and Tables

**Table 1:** Global Moran’s *I* test of wastewater discharge in China, 2002–2013.

Year	Moran’s *I*	*E*(*I*)	*Sd.*	*P*(*I*)
2002	0.2182	−0.0333	0.0109	0.0160
2003	0.2237	−0.0333	0.0106	0.0127
2004	0.2386	−0.0333	0.0109	0.0091
2005	0.2210	−0.0333	0.0105	0.0131
2006	0.2438	−0.0333	0.0105	0.0069
2007	0.2568	−0.0333	0.0105	0.0047
2008	0.2517	−0.0333	0.0108	0.0061
2009	0.2673	−0.0333	0.0108	0.0039
2010	0.2725	−0.0333	0.0108	0.0033
2011	0.2842	−0.0333	0.0106	0.0021
2012	0.2649	−0.0333	0.0104	0.0035
2013	0.2519	−0.0333	0.0104	0.0051

**Table 2:** The classification of the main driving factors affecting the provincial wastewater discharge in China (per one hundred million cubic meters).

The Classfication of the Main Driving Factors	Province	efc	tec	eco	pop	All
Two-factor dominant type	Jiangsu	6.79	54.68	53.71	2.01	117.19
	Guangdong	25.48	69.72	56.06	9.03	160.29
Three-factor leading type	Hubei	1.94	27.93	30.91	0.49	61.27
	Sichuan	3.59	29.04	31.22	0.21	64.06
	Zhejiang	6.53	30.19	29.46	3.01	69.19
	Hunan	4.59	30.5	30.76	1.47	67.32
	Shandong	13.49	29.79	33.72	1.74	78.74
	Henan	9.61	28.37	32.14	0.08	70.2
	Jiangxi	3.9	15.59	18.2	0.7	38.39
	Chongqing	1.86	17.88	18.07	0.82	38.63
	Liaoning	2.91	24.77	23.4	0.61	51.69
	Fujian	0.1	20.46	23.66	1.22	45.44
	Anhui	5.3	19.26	24.26	0.28	49.1
	Hebei	9.58	24.1	21.79	1.61	57.08
	Guangxi	5.39	24.51	26.43	0.00	56.33
Four-factorantagonistic type	Hainan	0.23	3.55	3.65	0.24	7.67
	Ningxia	0.12	3.74	4.23	0.28	8.37
	Qinghai	1.68	3.8	2.32	0.11	7.91
	Tibet	0.28	0.48	0.36	0.035	1.155
	Tianjin	1.43	7.36	6.22	2.23	17.24
	Xinjiang	1.62	6.29	7.43	0.81	16.15
	Gansu	2.84	6.5	5.49	0.08	14.91
	Yunnan	6.55	10.56	12.15	0.53	29.79
	Shanxi	3.58	12.74	13.49	0.19	30
	Inner Mongolia	3.33	9.2	10.08	0.28	22.89
	Guizhou	4.14	9.42	9.46	0.38	23.4
	Shanxi	2.35	10.51	10.8	0.87	24.53
	Beijing	3.49	10.76	7.78	3.45	25.48
	Jilin	0.47	9.35	11.78	0.11	21.71
	Shanghai	1.78	14.73	11.79	4.62	32.92
	Heilongjiang	9.28	16.84	11.23	0.042	37.392

Notes: efc represents the efficiency of the water resource utilization on the added value of wastewater discharge; tec represents technological advances on the added value of wastewater discharge; eco represents the economy of scale on the value added value of wastewater discharge; pop represents the population on the added value of wastewater discharge.
